# Supplementary material for: Structural and biochemical basis for activity of Aspergillus nidulans α-1,3-glucanases from glycoside hydrolase family 71
Source: Commun Biol. 2025 Aug 28;8:1298. doi: 10.1038/s42003-025-08696-3 (PMC12394665; doi:10.1038/s42003-025-08696-3)
Supplement: Supplementary file 1 — Supplemental Information [file 42003_2025_8696_MOESM1_ESM.docx]

**Supplemental information**

# Structural and biochemical basis for activity of *Aspergillus nidulans* α-1,3-glucanases from glycoside hydrolase family 71

Scott Mazurkewich^1,2^*, Tove Widén^1^, Hampus Karlsson^3,2^, Lars Evenäs^3,2^, Poornima Ramamohan^4,2^, Jakob Wohlert^4,2^, Gisela Brändén^5^, and Johan Larsbrink^1,2^*.

^1^ Department of Life Sciences, Chalmers University of Technology, Gothenburg, Sweden

^2^ Wallenberg Wood Science Center, Chalmers University of Technology, Gothenburg, Sweden

^3^ Department of Chemistry and Chemical Engineering, Chalmers University of Technology, Gothenburg, Sweden

^4^ FibRe-Centre for Lignocellulose-based Thermoplastics, Chalmers University of Technology, Gothenburg, Sweden

^5^ Department of Fibre and Polymer Technology, KTH Royal Institute of Technology, Stockholm, Sweden

^6^ Department of Chemistry and Molecular Biology, University of Gothenburg, Gothenburg, Sweden

* Correspondence should be addressed to either: [scott.mazurkewich@chalmers.se](mailto:scott.mazurkewich@chalmers.se) or [johan.larsbrink@chalmers.se](mailto:johan.larsbrink@chalmers.se)

**Supplementary Tables**

**Supplementary Table 1: Primers used for the amplification of genes used in the study and for the mutagenesis to generate the enzyme variants.** The mutations introduced by the primers are written in lowercase. The sequences for the 5’ overhang for ligation into pET-28a-TEVc by In-Fusion cloning (Takara Bioscience) are underlined.

| *Ss*GtfJ-f | CTTCCAGGGCCATAGTGATGAAACTCAAGATAAGACAGTAACTCAATCAAATTC |
| --- | --- |
| *Ss*GtfJ-r | TGGTGGTGCTCGAGTCTAGTTTAGCACTCTAGGTGGATAAGCAAGAC |
| *Ss*GtfL-f | CTTCCAGGGCCATAGTACTAACGACGGTGTCGTTCAGGC |
| *Ss*GtfL-r | TGGTGGTGCTCGAGTCTAAATTTGACCTCGGCCTTCACCGTC |
| *An*GH71B-f | CTTCCAGGGCCATAGTGCCTTCCCGCAGCTCAAG |
| *An*GH71B-r | TGGTGGTGCTCGAGTCTACTATGCAGTAGGCTGTGAAGCATTG |
| *An*GH71B-D271A-r | CTCCGTAAgCGTTCCAGGTAACGATTTC |
| *An*GH71B-D271A-f | CTGGAACGcTTACGGAGAGTCGC |
| *An*GH71B-E274A-r | GTGCGACgCTCCGTAATCGTTCC |
| *An*GH71B-E274A-f | CGGAGcGTCGCACTACATTGGAC |
| *An*GH71C-f | CTTCCAGGGCCATAGTCTACCGGGAGCGAACAGTCTC |
| *An*GH71C-r | TGGTGGTGCTCGAGTCTACTAGGCGCTAAAAGAGCCAACATAAAC |
| *An*GH71C-D265A-f | CTGGAATGcCTACGGCGAGTCC |
| *An*GH71C-D265A-r | GCCGTAGgCATTCCAGGAAATGATCTC |
| *An*GH71C-E268A-f | CTACGGCGcGTCCCACTACATC |
| *An*GH71C-E268A-r | GTGGGACgCGCCGTAGTCATTC |

**Supplementary Table 2: The gradient used in the method for separation of nigerooligosaccharides.** The eluents used were deionized water (Eluent A), 300 mM NaOH (Eluent B) and 1 M sodium acetate (NaAc) + 100 mM NaOH (Eluent C) all sparged with nitrogen for removal of dissolved oxygen. The flow was constant at 0.5 ml/min throughout the method. First the system was equilibrated with 15% eluent B for 5 min before the injection at timepoint 0. A linear gradient increasing from 15% to 33% for eluent B and from 0% to 15% for eluent C is then applied over 20 min. The column is subsequently cleaned with 33% eluent B and 66% eluent C for 2.5 min and then equilibrated with 15% eluent B for 2.5 min. A representation of the elution protocol can be found below the table.

| Time period (min) | Eluent B (%)  300 mM NaOH | Eluent C (%)  1M NaAc + 100 mM NaOH |
| --- | --- | --- |
| -5 – 0 | 15 | 0 |
| 0 – 20 | 15 to 33 | 0 to 15 |
| 20 – 22.5 | 33 | 66 |
| 22.5 – 25 | 15 | 0 |


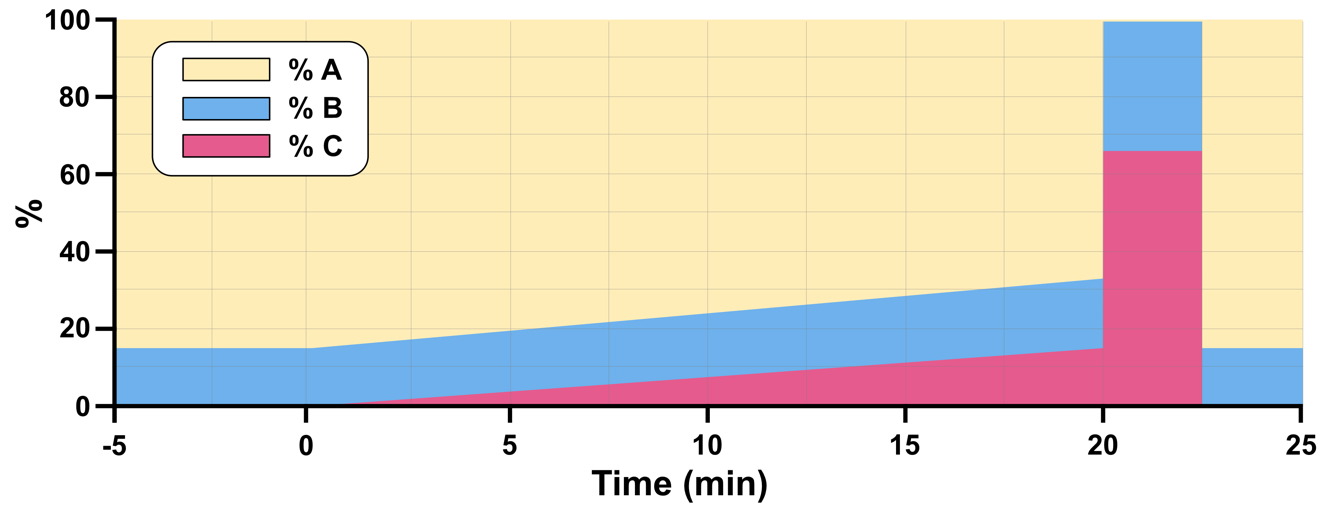


**Supplementary Table 3: Composition of modified Czapek-Dox media.** Note that stock solutions of ^13^C glucose and ^15^N sodium nitrate were prepared, filter sterilized through a 0.2 μm filter, and added to the other media components after they had been sterilized by autoclaving.

| **Component** | **Concentration (g/L)** |
| --- | --- |
| Dipotassium phosphate | 1.0 |
| Magnesium sulphate | 0.5 |
| Potassium chloride | 0.5 |
| Iron sulphate | 0.1 |
| Sodium nitrate | 2.0 |
| Glucose | 10 |

**Supplementary Table 4:** **Conditions used for protein crystallisation.** For ligand complexes, crystals were soaked in reservoir solution containing a saturating amount of ligand for 2 min prior to flash freezing in liquid nitrogen.

| **Protein-Ligand** | **Crystallisation condition** |
| --- | --- |
| *An*GH71C | 0.1 M BIS-Tris pH 5.5 with 0.2 M ammonium sulphate and 25 % w/v PEG 3350 |
| *An*GH71C-Glucose | 0.1 M Citrate pH 5.0 with 20 % w/v PEG 6000 |
| *An*GH71C-Nigerotetraose | 0.1 M Citrate pH 5.0 with 20 % w/v PEG 6000 |

**Supplementary Figures**


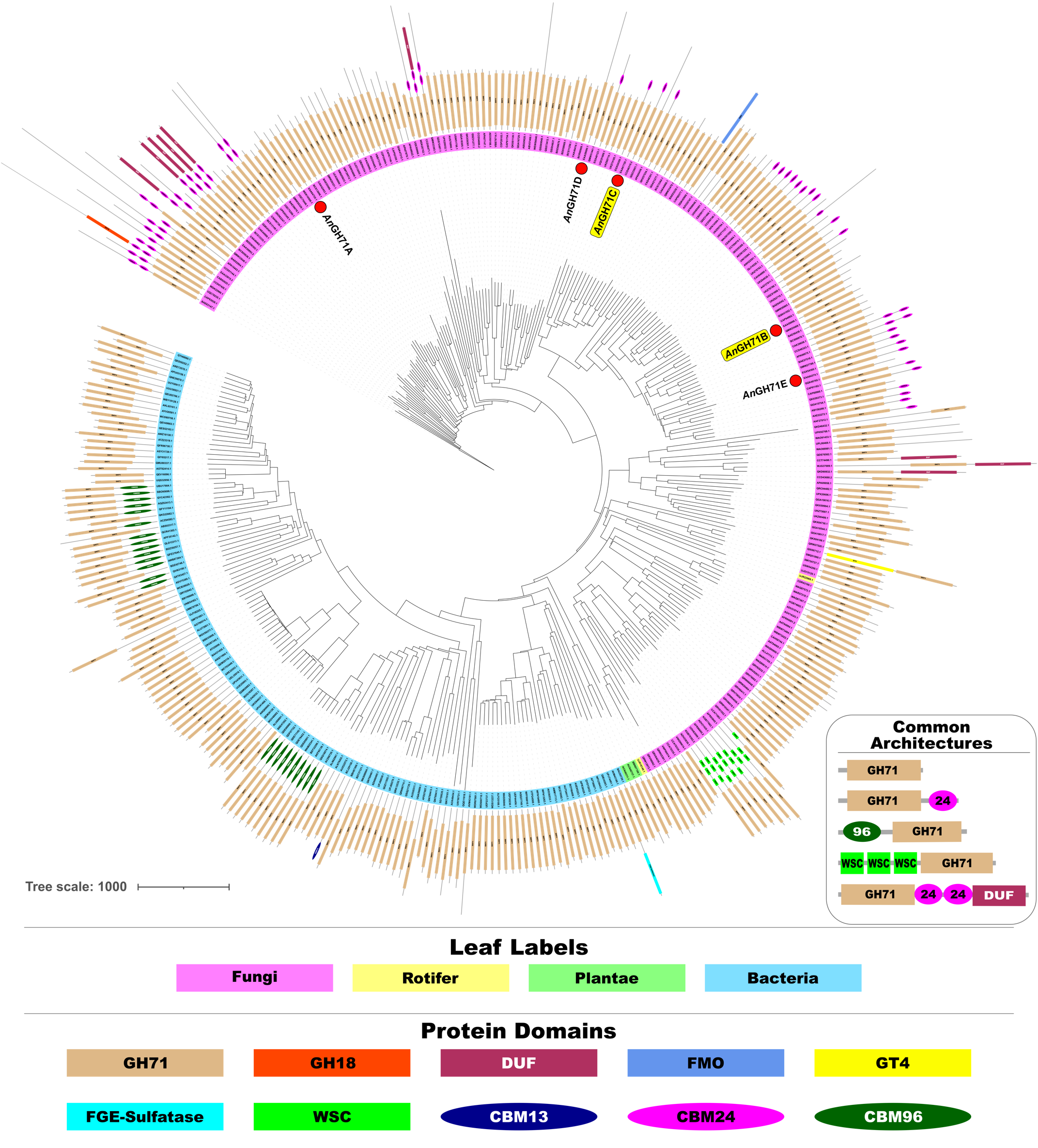


**Supplementary Fig. 1: Expanded phylogenetic tree of GH71 with protein architectures.** The tree of the GH71 domain was created with iTOL using curated sequences from CAZy and domain annotations as described in the methods. Entries are labelled with their Genbank identifier, and the leaf labels are coloured with the proteins origin: fuchsia for fungal, blue for bacterial members, green for plantae, and yellow for the entry from the rotifer *Adineta vaga*. Enzymes from *A. nidulans* FGSC A4 are identified by a red circle with both *An*GH71B and *An*GH71C additionally highlighted in yellow. The architecture of each gene product is shown radially from the leaf and additional protein domains found with GH71 members include: glycoside hydrolase family 18 (GH18), a domain of unknown function (DUF) found conserved amongst several members, flavin-binding monooxygenase (FMO), glycosyltransferase family (GT4), formylglycine-generating enzyme (FGE)-sulfatase, carbohydrate modules from families 13 (CBM13), 24 (CBM24), and 96 (CBM96), and cell wall integrity and stress response component (WSC) domains which could also function as CBMs. The common architectures, defined as observed > 3 times, are listed in the inset. A pdf file and a vector format for this figure is supplied as an additional file which can be used for searching of accessions.


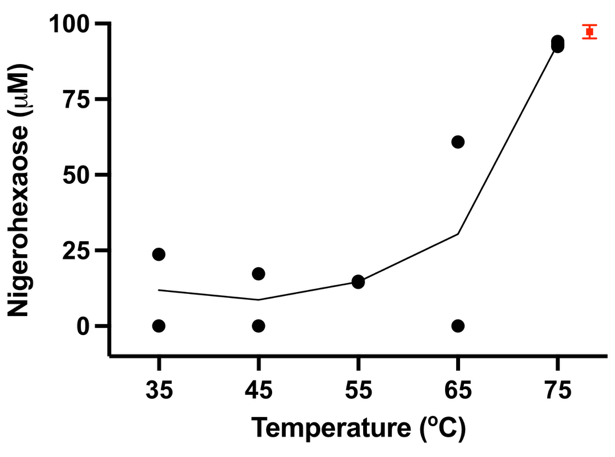


**Supplementary Fig. 2: Stability of *An*GH71C at different temperatures.** Stability of the protein was assessed by heat treatment of the enzyme at different temperatures for 2 min, followed by cooling on ice for 2 min, and subsequently subjected to centrifugation at 21,000 × g for 2 min. The heat-treated enzymes were then assayed with nigerohexaose by the standard methodology described in the methods section and the amount of the nigerohexaose remaining in solution was quantified by HPAEC-PAD and compared to a no enzyme control (red). Data points are from duplicate assays (n = 2).


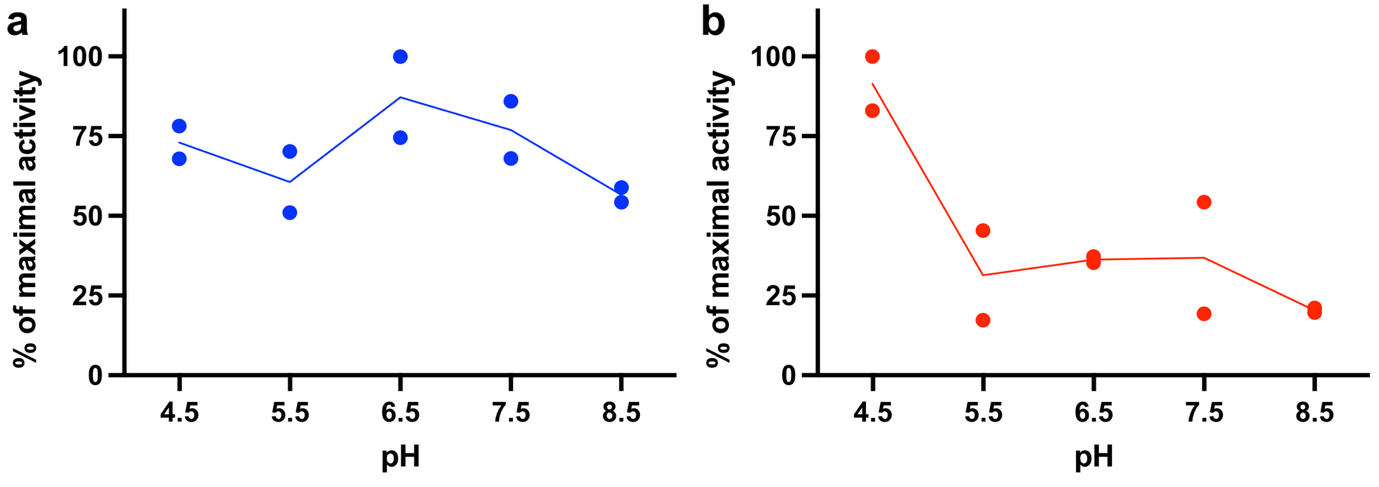


**Supplementary Fig. 3: Effect of pH on hydrolase activity.** The specific activity of a) *An*GH71B (blue) and b) *An*GH71C (red) with 100 μM nigeropentaose at different pH values was measured in duplicate, with the specific activity of each run plotted as relative activity to the activity in pH giving the maximal activity observed for each enzyme.


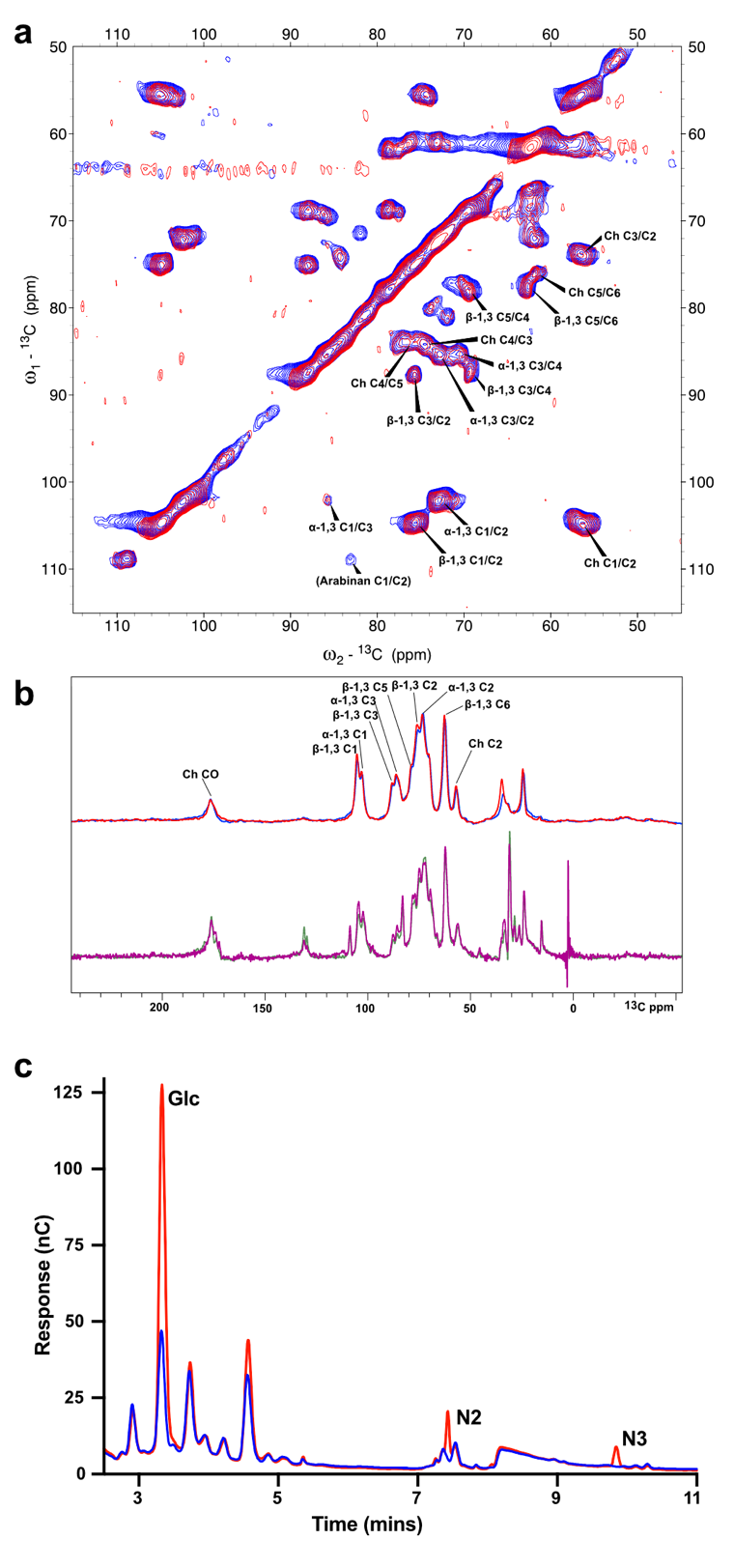


**Supplementary Fig. 4: Treatment of *Aspergillus nidulans* biomass with *An*GH71C.** a) 2D ^13^C,^13^C-RFDR correlation spectra showing short range correlations of rigid cell wall components of *A. nidulans* before (blue) and after (red) treatment with 0.5 nmol of *An*GH71C. Indicated assignments refer to α-1,3-glucan, β-1,3-glucan and chitin. b) Superimposed 1D ^13^C spectra of intact *A.* *nidulans* biomass. The two upper spectra are CP-MAS spectra before (blue) and after (red) enzyme treatment, signals clearly belonging to specific cell wall components are indicated. The two lower spectra are direct polarization spectra before (green) and after (purple) enzyme treatment. c) Soluble extracts from ball-milled *A. nidulans* before (blue) and after (red) overnight treatment with 0.5 nmol of *An*GH71C, detected by HPAEC-PAD, which results in liberated glucose (Glc), nigerose (N2), and nigerotriose (N3).


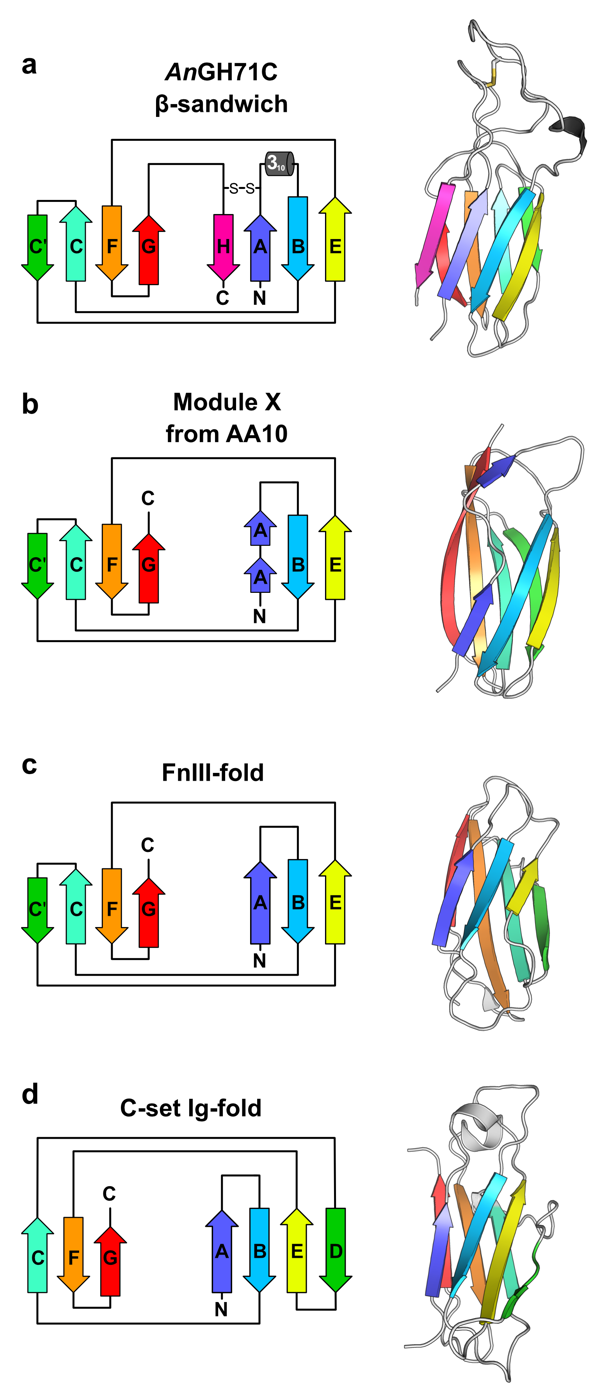


**Supplementary Fig. 5: Relation of the *An*GH71C C-terminal β-sandwich domain to homologs.** The topology and structures of the a) *An*GH71C C-terminal β-sandwich domain, b) module X from *Vc*LPMO10B (previously referred to as GbpA, PDB accession 2xwx), c) the third FnIII domain from human tenascin (PDB accession: 1ten), and d) the C1-set immunoglobulin (Ig) fold of a mouse IgG2a (PDB accession: 1igt). The β-strands are designated based on the convention of Ig-folds where βD packs with βE and the additional strands before that are designated as C’. The disulphide bond in *An*GH71C linking the long loops between βA-βB and βG-βH is shown as sticks in the right panel.

**
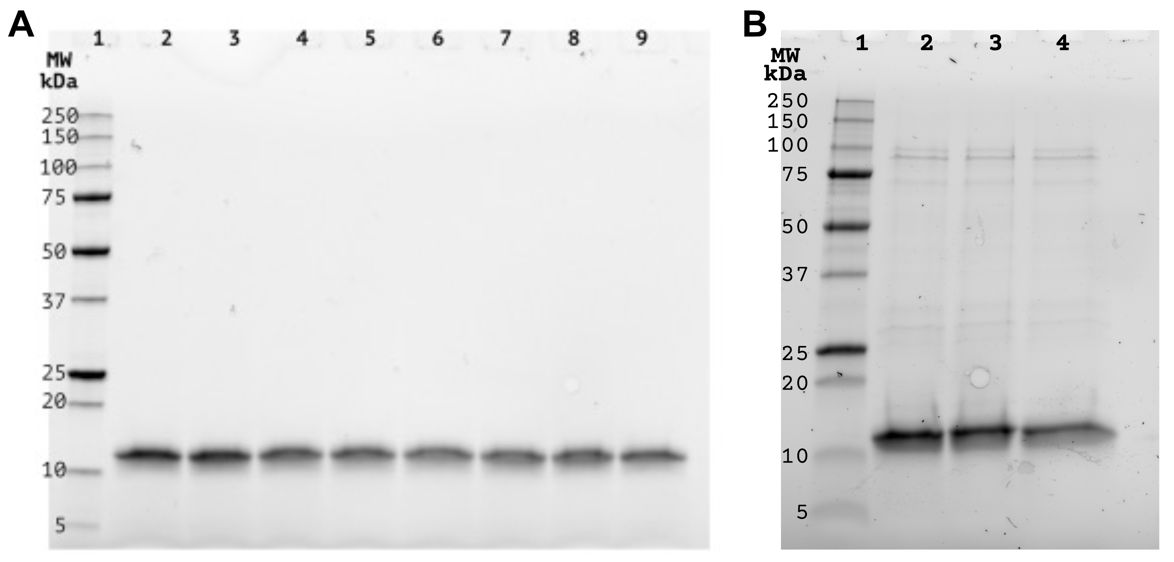
**

**Supplementary Fig. 6: SDS-PAGE analysis of the pull-down assay of the C-terminal domain of *An*GH71C with insoluble polysaccharides.** Assays of 200 uL were completed by mixing protein (0.3 mg/mL) with polysaccharide (1.0 mg/mL) in 50 mM Tris pH 8 containing 250 mM NaCl for 1 h at room temperature with constant agitation after which the insoluble material was removed by centrifugation at 10,000 ×g for 2 min. Soluble portions were loaded into 10% Mini-PROTEAN® TGX Stain-Free™ Protein Gels (Bio-Rad), resolved, and visualized by stain-free methodologies (as described by the manufacturer). In panel A, lanes show the 1) molecular weight ladder and the C-terminal domain remaining in solution after being mixed with either 2) control with no polysaccharide, 3) mutan, 4) alternan (α1,3/α1,6-glucan), 5) nigeran (BioSynth), 6) potato starch, 7) Avicel, 8) cellulose microgranules, or 9) xylan from beech wood. In panel B, lanes show the 1) molecular weight ladder and the C-terminal domain remaining in solution after being mixed with either 2) control with no polysaccharide, 3) ball milled α-chitin, or 4) ball milled β-chitin.

**
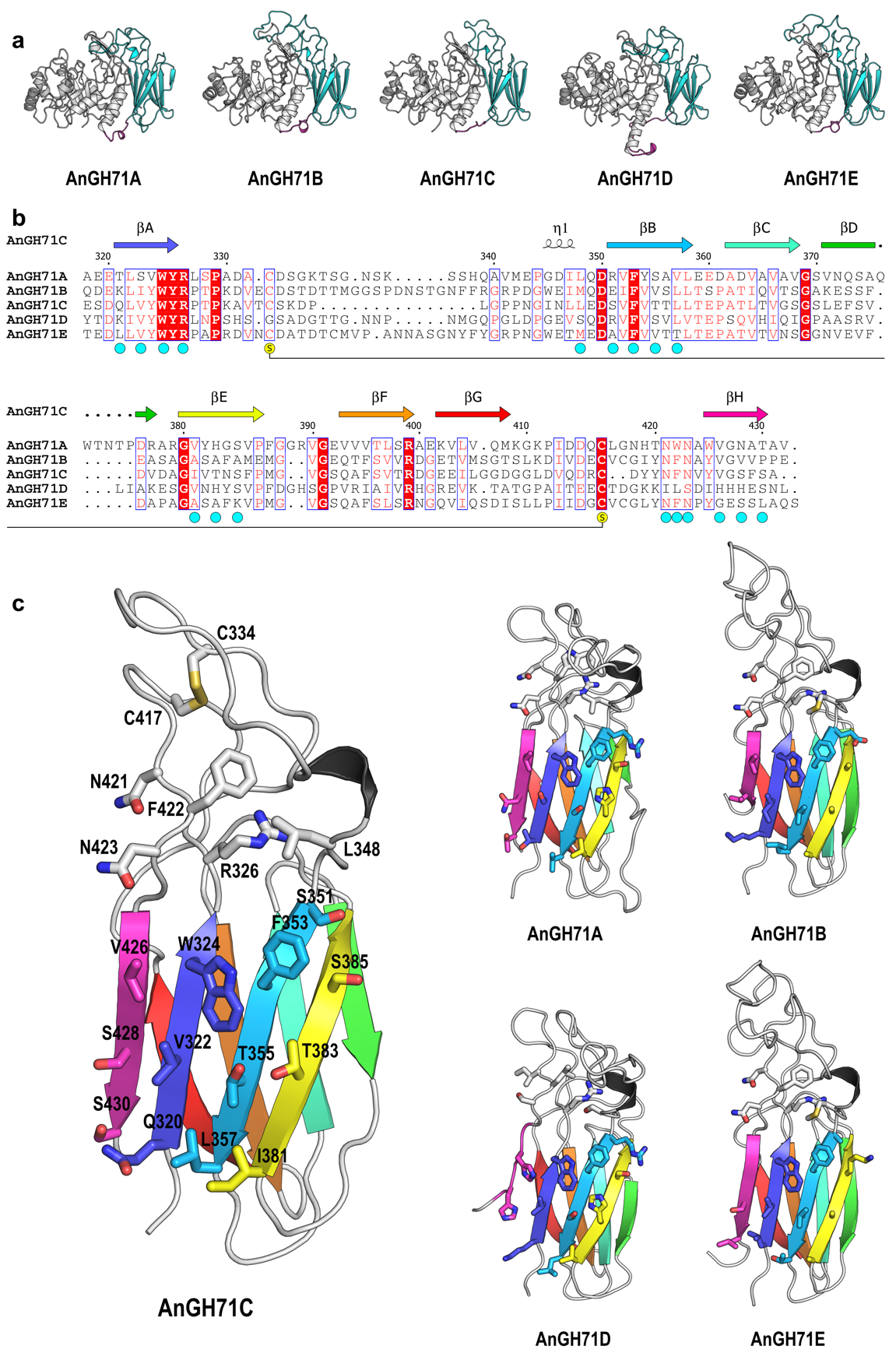
**

**Supplementary Fig. 7: Comparison of the C-terminal β-sandwich domain amongst the GH71 members from *A. nidulans*.** a) Overall structures of the GH71 domains of *An*GH71A, -B, -D, and -E generated by AlphaFold2^1, 2^ and the experimentally determined *An*GH71C showing a similar overall structure with a N-terminal (β/α)_8_-barrel (grey), linker (magenta), and C-terminal β-sandwich domain (cyan). b) Sequence alignment of the C-terminal β-sandwich domain with the secondary structure of *An*GH71C shown above the alignment with the strands coloured in rainbow. The cysteine residues forming a disulphide bond in *An*GH71C are denoted below the alignment by a yellow circle. Some of the residues whose sidechains line the face of the sandwich and pack against the N-terminal (β/α)_8_-barrel are identified by cyan spheres below the alignment. c) Structures of the C-terminal β-sandwich domain with the side chains of the residues highlighted in the sequence alignment shown as sticks.


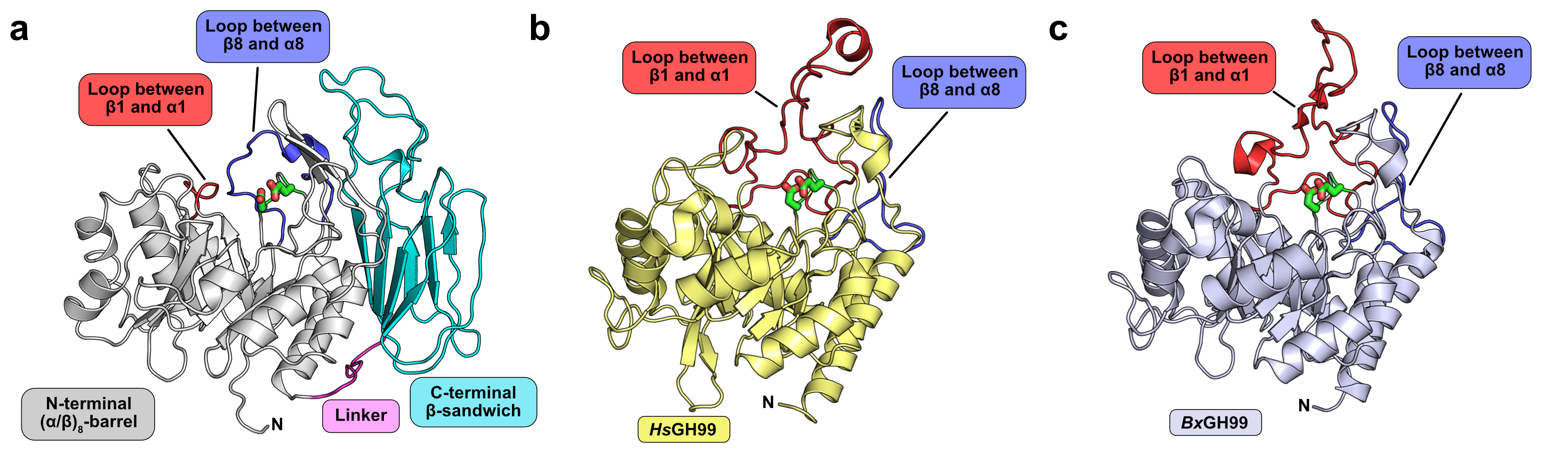


**Supplementary Fig. 8: Comparison of the overall structure of *An*GH71C with GH99 members.** a) Overall structure of *An*GH71C coloured as in Fig. 5 with additional colouring of the loops between β1-α1 and β8-α8 in red and blue, respectively. The overall structures of GH99 members from (b) *Homo sapiens* (*Hs*GH99, PDB accession: 6zdf) and (c) *Bacteroides xylanisolvens* (*Bx*GH99, PDB accession: 4ad1) with their loops between β1-α1 and β8-α8 similarly coloured as *An*GH71C. In all, the conserved active site acidic residues are shown as green sticks.


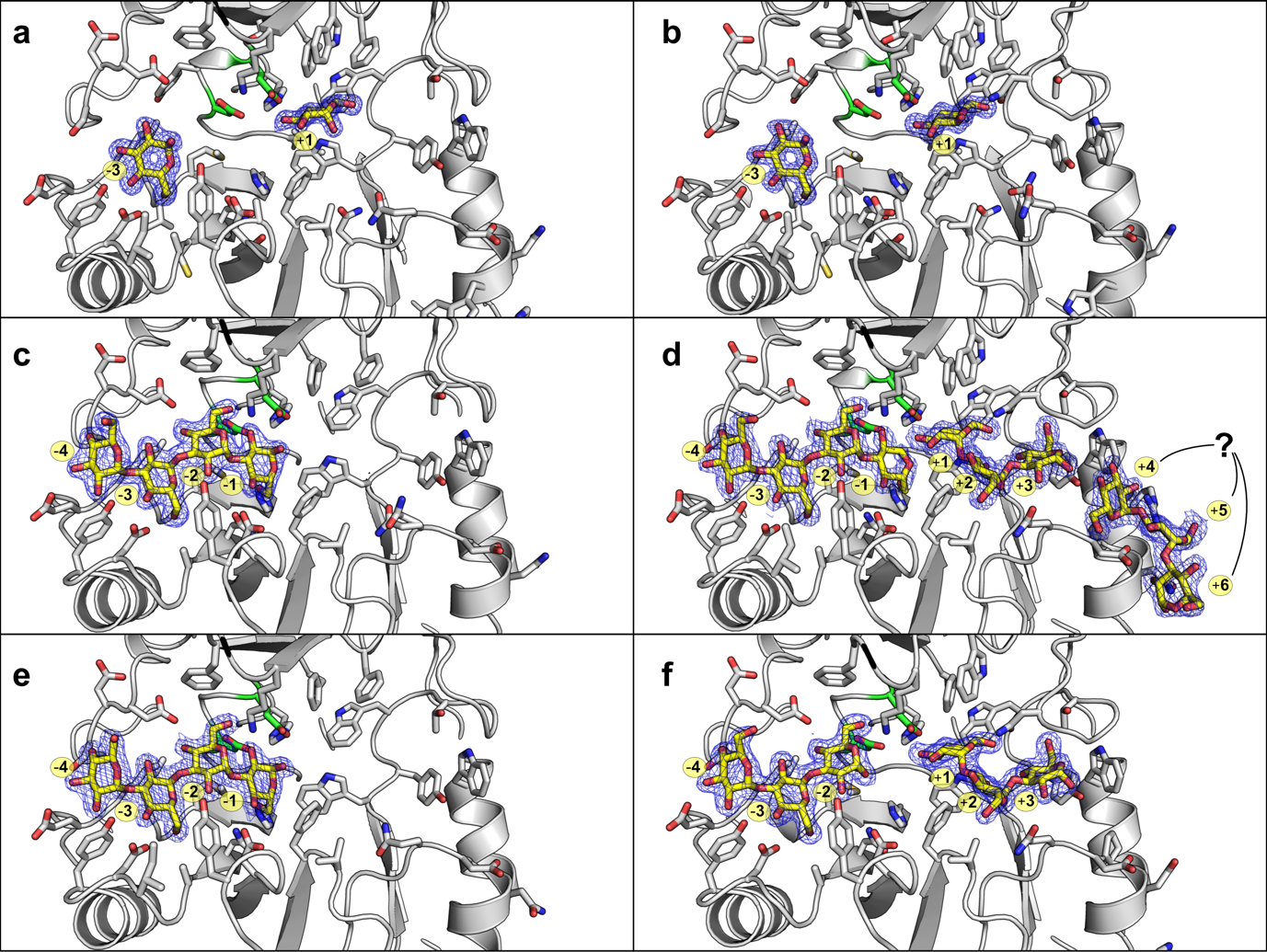


**Supplementary Fig. 9: Ligand complexes of *An*GH71C.** The two catalytic acidic residues are coloured green, the ligands and subsite numbers in yellow, and electron density from the 2Fo – Fc map for the ligands contoured to 1.25 σ is shown in blue. The two *An*GH71C chains in complex with glucose (a and b) reveal glucose molecules bound in the same -3 and +1 sites in both chains. The nigerotetraose soaked crystal was of space group P1 and contained four protein chains in the asymmetric unit (c, d, e, and f). In the minus subsites, nigerotetraose was observed in the -4 to -1 sites in chains A, B, and C (panels c, d, and e) while nigerotriose was observed in the -4 to -2 subsites in chain D. No ligand was observed with in the plus subsites of chains A and C (panels c and e) while nigerotriose was observed in the +1 to +3 subsites in both chains B and D (panels d and f). Nigerotriose was also observed on the surface of the cleft in chain B (panel d) extending from the +3 subsite and could be bound in +4, +5, and +6 subsites however the trisaccharide makes few direct interactions with the protein making the subsites annotations tentative and is marked with a question mark.


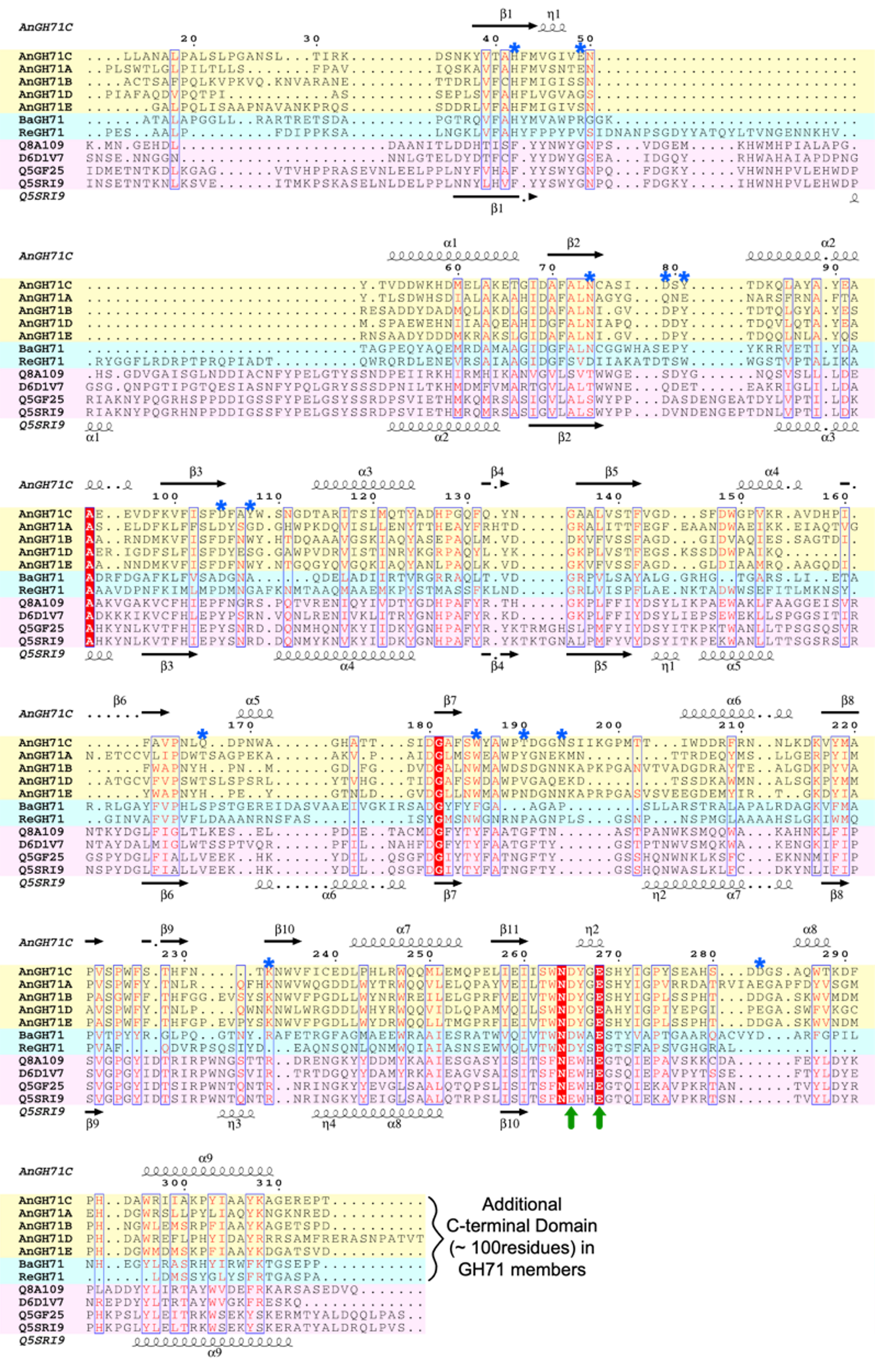


**Supplementary Fig. 10: Sequence alignment of the N-terminal (β/α)_8_-barrel domain of select GH71 proteins with select characterized GH99 members.** Sequences included are the five enzymes from *Aspergillus nidulans* (*An*GH71A-E), bacterial GH71 members from *Burkholderia ambifaria* AMMD ATCC BAA-244 (*Ba*GH71) and *Rhodococcus erythropolis* NBRC 100887 (*Re*GH71), and GH99 members from *Bacteroides thetaiotaomicron* VPI-5482 (Q8A109), *Bacteroides xylanisolvens* XB1A (D6D1V7), *Rattus norvegicus* (Q5GF25), and *Homo sapiens* (Q5SRI9). The fungal GH71 members are highlighted in yellow, bacterial GH71 members in blue, and the GH99 members in pink. Identical residues are coloured white on a red background and similar residues coloured red boxed in a blue box. The catalytic acidic residues conserved amongst the GH71 and GH99 members are indicated by green arrows below the alignment. Note that in GH71 members it is a DxxE dyad while in GH99 members it is a ExxE motif. GH71 members contain an additional C-terminal domain that is not shown in this alignment. The secondary structural elements of *An*GH71C and the GH99 from *Homo sapiens* (PDB accession: 6ZDF) are shown above and below the alignment, respectively. Residues whose sidechains are observed making direct interaction with the nigerooligosaccharides are indicated by a blue asterisk above the alignment.


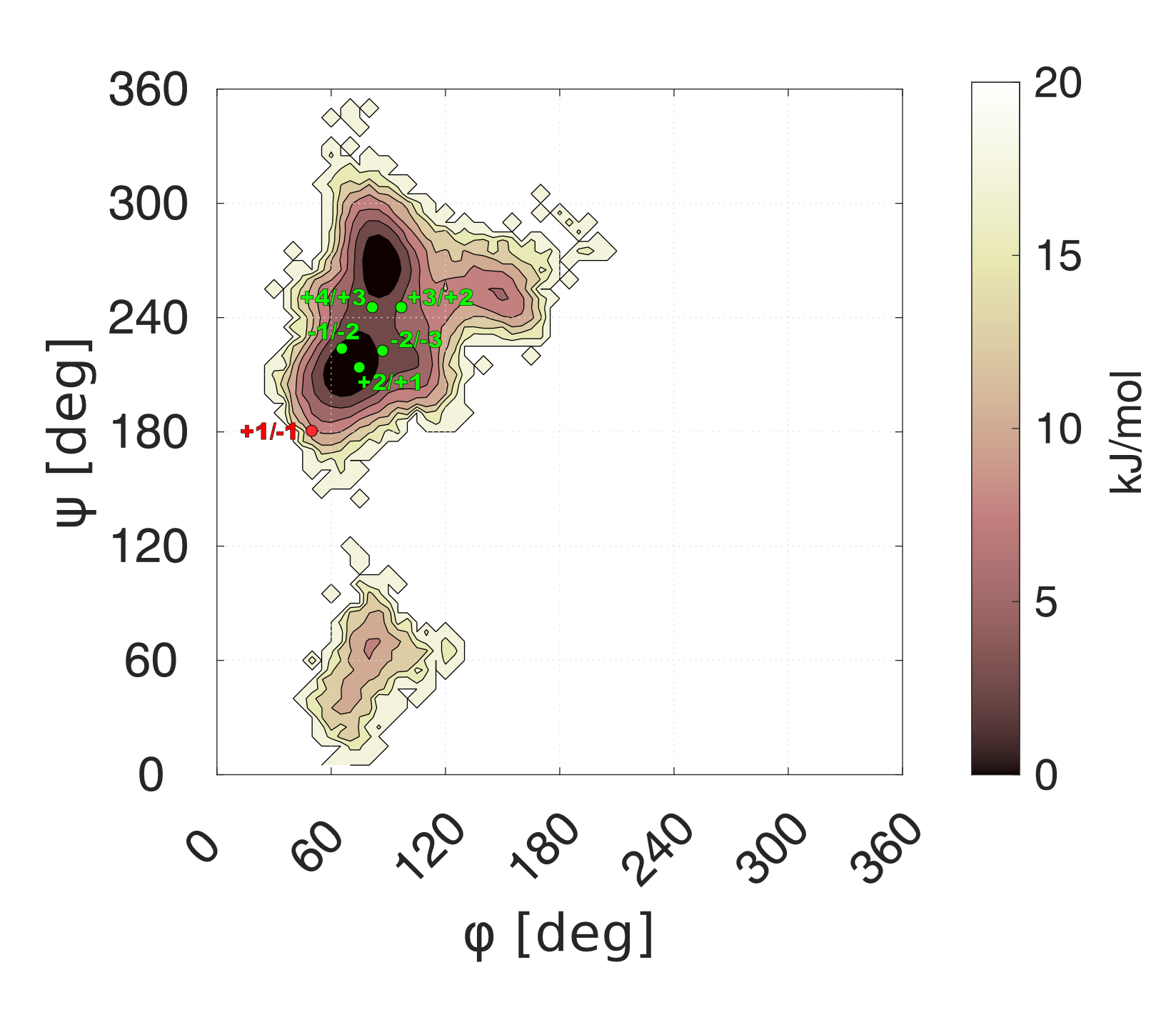


**Supplementary Fig. 11: Comparison of the glycosidic bond angles of the ligands observed in the *An*GH71C complex structure to *in-silico* simulations of nigerohexaose.** The dihedral angles of the glycosidic linkages $\varphi$ (O5-C1-O3’-C3’) and $\psi$ (C1-O3’-C3’-C2’) were sampled from a 100 ns long replica-exchange molecular dynamics simulation of nigerohexaose in water and saved as a 2D histogram, which was converted into an energy contour map through Boltzmann inversion. The energy scale was set relative the most probable conformation. The linkages between sugar units in the ligand complex structure are shown in spheres with the linkage across the cleavage site coloured in red and all others in green. Notably, the ligands in the binding subsites occupy preferred conformations while the linkage angles of the molecule across the cleavage occupies a comparatively higher energy conformation when it is primed for catalytic cleavage.


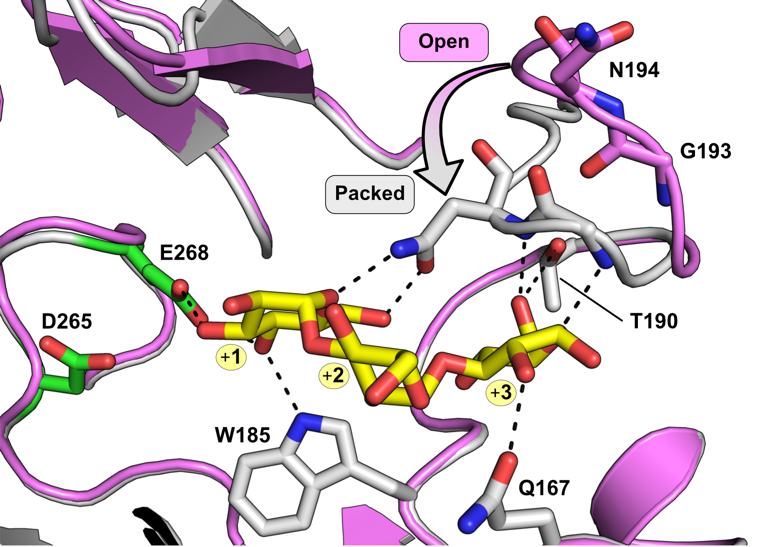


**Supplementary Fig. 12: Movement of the loop between β6 and α6 in *An*GH71C upon ligand binding.** Upon binding of ligand in the +1 to +3 site the loop between β6 and α6 moves from an open state (light pink) to pack on top of the ligand leading to interactions with Gly193 and Asn194. Hydrogen bonds (defined as electrostatic interactions ≤3.2 Å) are shown with black dashes.


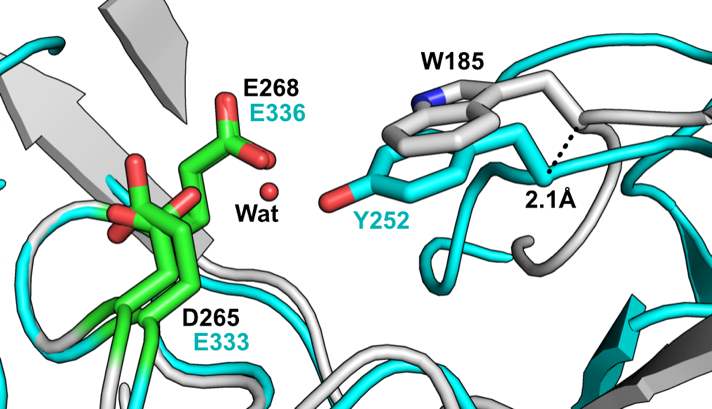


**Supplementary Fig. 13: Comparison of the catalytic sites of *An*GH71C and *Bx*GH99.** The region around the catalytic site of *An*GH71C (grey) and *Bx*GH99 (cyan; PDB accession: 6fwp) with the residues of the catalytic dyad shown in green for both. Relative to *Bx*GH99, the distance (2.1 Å) resulting the further set back loop in *An*GH71C is shown which contributes, in addition to the difference in aromatic side chain identity, to the creation of a small gap for the active site water, and likely catalytic nucleophile, to be bound.


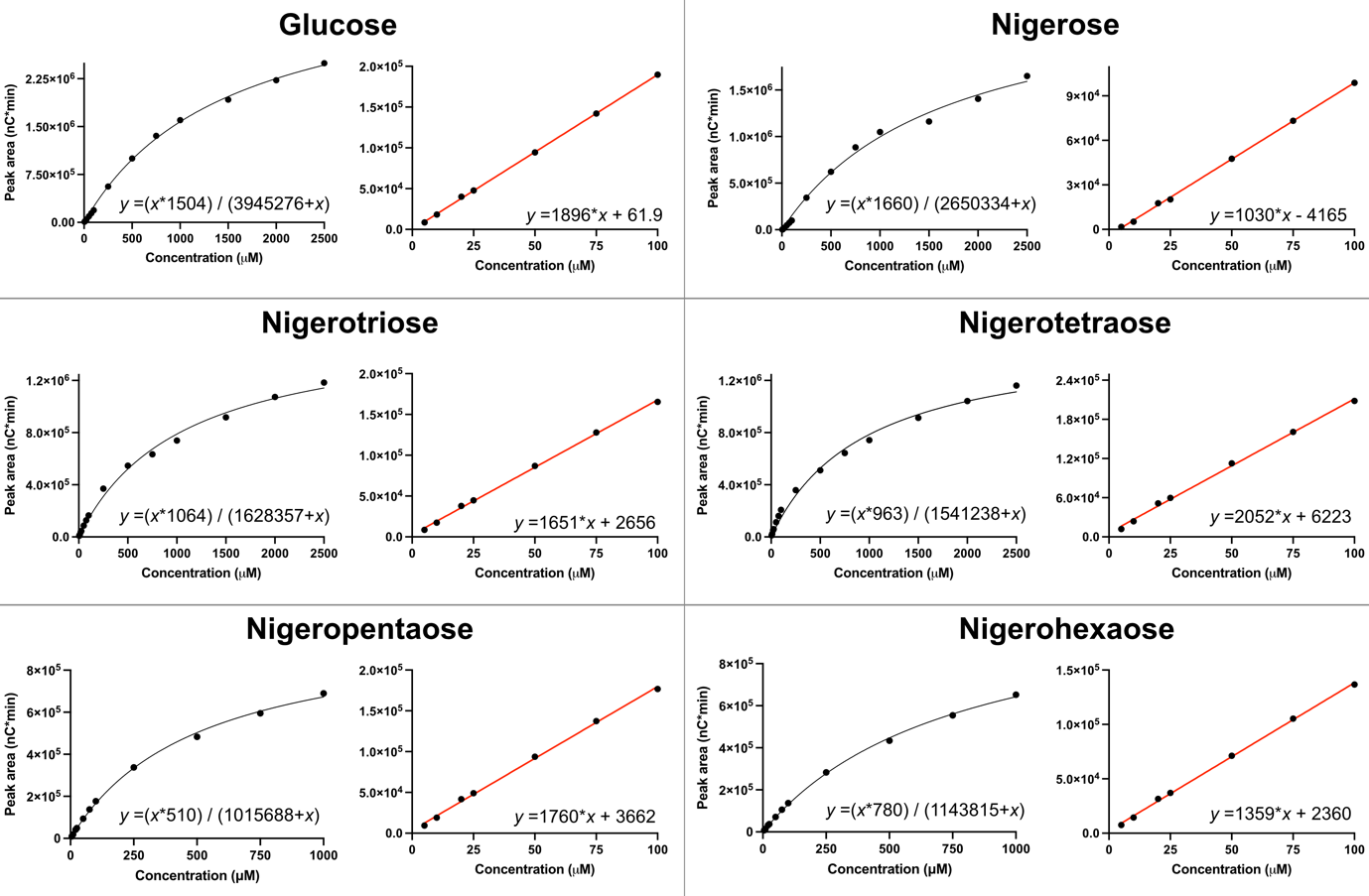


**Supplementary Fig. 14: Standard curves of nigerooligosaccharides determined by HPAEC-PAD.** The peak area of oligosaccharide standards fit by non-linear regression to the saturation equation (Y = B_max_*X/[K + X]) for concentrations between 5 to 2500 μM or linear regression for concentrations between 5 to 100 μM. Note that the quantification of oligosaccharides by non-linear regression was only utilized in the mutan degradation assays presented in Figures 2b and d.

**Supplementary References**

1. Jumper J*, et al.* Highly accurate protein structure prediction with alphafold. *Nature* **596**, 583-589 (2021).

2. Varadi M*, et al.* Alphafold protein structure database: Massively expanding the structural coverage of protein-sequence space with high-accuracy models. *Nucleic Acids Res* **50**, D439-D444 (2022).
